# Meigs' Velpeau's Midwifery

**Published:** 1852-04

**Authors:** 


*Meigs' Velpeau's Midwifery.* Fourth American, with the additions from the last French edition by W. Byrd Page, M. D. &c., pp. 652. Philadelphia: Lindsay & Blakiston, 1852.

To commend Velpeau's Midwifery, would be to "gild refined gold or paint the lily." Its character has long since been established as a standard book on obstetrics. Dr. Meigs' translation has also gone through four editions, a sufficient evidence of its acceptability.

The engravings in this edition are numerous and well executed, and will much facilitate the acquirement of correct information. The lines have fallen to the modern student on pleasant places. It is easy now to master the mysteries which once so tried the patience and perseverance of us elders, when in our younger days we pored over the solid pages, unbroken by a cut, and tried in vain to form images in our minds of the things described.

We can honestly recommend this book. Midwifery should be understood thoroughly by every man who undertakes to practice it, and to understand it thoroughly, it is necessary to read Velpeau.

